# Infrared Spectra of Asphalts

**DOI:** 10.6028/jres.063A.014

**Published:** 1959-10-01

**Authors:** Burton D. Beitchman

## Abstract

A procedure for preparing thin films of airblown asphalts and a spectroscopic study of such films throughout the infrared region of 2.5 to 15 microns are described. The relationship of transmittance of several absorption bands with the durability of the asphalt is discussed. Exposure to ultraviolet radiation produced changes in transmittance at four wavelengths which, in a series of asphalts studied, showed statistically significant correlations with durability.

## 1. Introduction

Roofing asphalts vary greatly in durability depending upon the source of the particular asphalt. One method for measuring durability that is currently being used is based upon the time required for development of cracks over 50 percent of the surface of an asphalt film when such a film is exposed to natural weathering conditions or to radiation from a carbon arc, in an accelerated weathering test.^1^

Stewart 2 recently reported an infrared study of components from three asphalts before and after natural weathering. These asphalts varied in durability, and Stewart pointed out chemical properties in the components that might be associated with the durabilities of the asphalts.

An infrared study of the whole asphalt appeared desirable since the general characteristics of asphalts could be readily indicated without resorting to a fractionation procedure. The use of whole asphalt permits easy study of changes effected in the asphalt by exposure to heat, ultraviolet, and other forms of radiation.

Schweyer 3 has reported a study of films of asphalt residua. These films were too thick (0.1 mm) to permit study below 8 *μ* and he therefore resorted to solution techniques to obtain absorption data in this region.

The procedure described in this paper offers a simple means for studying whole asphalt.

## 2. Procedures

An infrared study was made of 28 airblown roofing asphalts from widely different sources. The softening points 4 of these asphalts ranged from 205° F to 241°F and the penetrations^5^ at 77° F from 11 to 23.

Films of asphalt were prepared by placing a small drop of molten asphalt on a sheet of cellophane film. A second sheet of cellophane was placed over the asphalt, and the two sheets were then quickly placed in a hydraulic press in which the upper platen was maintained at approximately 90° C.^1^,^6^ A pressure of 16,000 psi was applied to the sheets and maintained for about 20 sec. The sheets were removed from the press, and the thickness of the asphalt film and two cellophane sheets was measured with a dial-thickness gage having a foot about ¼ in. in diameter with which thickness could be estimated to the nearest 0.01 mil. By determining the thickness of the two cellophane sheets, the thickness of the asphalt film was obtained by difference. To ensure greater reproducibility of results, areas of nearly uniform thickness were predetermined and indicated by marking the boundaries of these areas on the cellophane film. The asphalt films were obtained in various thicknesses ranging from about 0.5 to 2.5 mils. By allowing the asphalt film and two sheets of cellophane to soak in distilled water for several minutes, one of the cellophane sheets could be removed easily. The asphalt film and the remaining cellophane sheet were soaked again in distilled water for several minutes in order to remove the second cellophane film. The asphalt film was mounted on a film support made of cardboard or plastic which had a rectangular opening, 1.4×3.5 cm. The predetermined area of nearly uniform thickness was placed over the opening. After allowing the asphalt films to dry, infrared spectra were taken.

The films prepared by this method were not perfectly uniform in thickness and thickness ranges have been recorded in this paper rather than average values. This must be considered a limitation where the transmittance (*τ*)^7^ of the unexposed films have been used for quantitative comparisons. The changes produced at four wavelengths by exposure to ultraviolet radiation did not appear to be dependent upon film thickness over the range of thickness employed. Thus, correlations which were made of changes in absorption with durability do not have this limitation imposed upon them.

## 3. Results

The infrared spectra of films of asphalts obtained from widely different sources showed a marked similarity in their absorption characteristics. All the asphalts had absorption bands at the same or very nearly the same wavelengths and their spectra differed primarily in the transmittance values of some of these bands.

The structural groupings which appeared to be present in all the asphalts examined are hydroxyl, –CH_2_–, C–CH_3_, carbonyl, –(CH_2_)_4_– and aromatic rings. There were also absorption bands which indicated aromatic substituents or fused rings. Other weak but reproducible bands were noted at 8.66 *μ* (1,155 cm^−1^) and 9.71 *μ* (1,030 cm^−1^). The structural assignments given to the various bands are listed in table 1. These are for the most part, the same groupings identified by Stewart^2^ in fractions of three asphalts.

**Figure 1:**
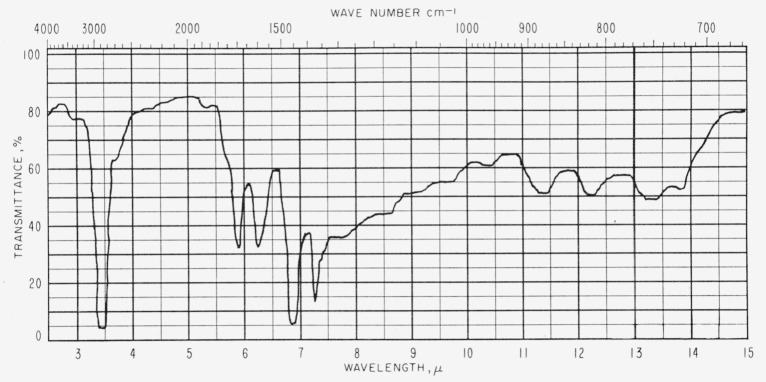
Infrared spectrum of asphalt *D* (1.8 to 2.1 mils thick).

**Figure 2:**
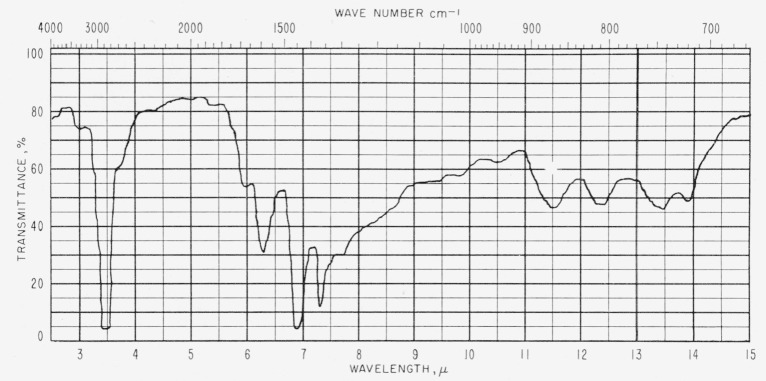
Infrared spectrum of asphalt *B* (1.9 to 2.3 mils thick).

Figures 1 and 2 show representative spectra of two of the asphalt films. Table 1 gives values of log 1/*τ*, determined by the base-line method, for absorption bands from the spectra of seven asphalts. The less durable asphalts appear to have slightly more hydroxyl absorption than the more durable asphalts. The C–CH_3_ band intensity at 7.25 *μ* (1,380 cm^−1^) seems also to show a relationship to durability. Uncertainties in the average thickness produce corresponding uncertainties in the transmittance. In the case of the hydroxyl band at 2.91 *μ* (3,430 cm^−1^), the uncertainties in average thickness do not invalidate the observed general correlation with durability. However, in the case of the band at 7.25 *μ* (1,380 cm^−1^), uncertainties in the value of log 1/*τ* may be sufficient to obscure any correlation with durability.

**Figure 3:**
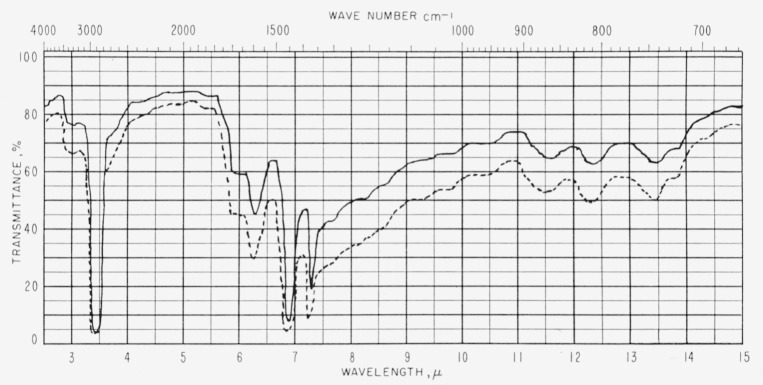
Infrared spectra of asphalt *E*. —— 1.1 to 1.3 mils thick, --- 1.8 to 2.0 mils thick.

Duplicate samples of asphalts A and F are included in table 1 to demonstrate the reproducibility of the spectra. The relation of film thickness to absorption intensities appears to follow the Beer-Lambert Law as illustrated in figure 3.

**Figure 4:**
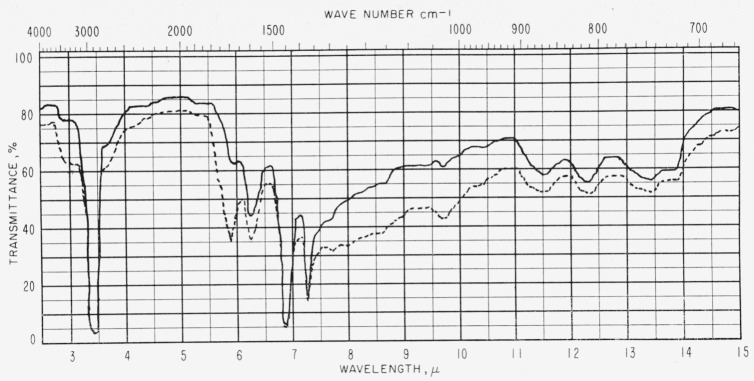
Infrared spectra of asphalt *U* (1.15 to 1.55 mils thick). —— before exposure, - - - after exposure.

**Figure 5:**
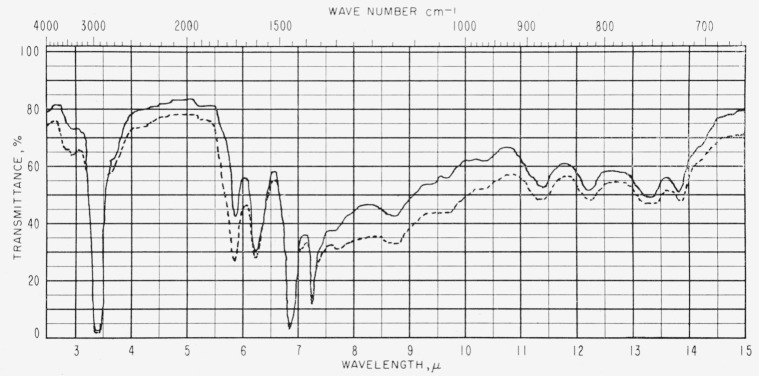
Infrared spectra of asphalt *V* (1.81 to 2.09 mils thick). —— before exposure, - - - after exposure.

Figures 4 and 5 show spectra of two asphalt films before and after exposure to radiation from a carbon arc for 19.5 hr. These spectra show characteristic changes that were also observed in other asphalts. Table 2 shows some of the changes produced in the values of log 1/*τ* of four asphalts. Changes in the values of log 1/*τ* for bands at 3.38 *μ* (2,960 cm^−1^) and 6.80 *μ* (1,470 cm^−1^) are not shown in table 2 (the films used for illustrative purposes in this table were too thick to show the changes in these regions). All the asphalts increased in carbonyl and hydroxyl absorption and the less durable asphalts generally showed a greater increase in carbonyl and hydroxyl absorption and absorption at 8.66 *μ* (1,155 cm^−1^) and 9.71 *μ* (1,030 cm^−1^) than the more durable asphalts.

Plots of durability values versus changes in log 1/*τ* at 5.88 *μ* (1,700 cm^−1^), 2.91 *μ* (3,430 cm^−1^), 8.66 *μ* (1,155 cm^−1^), and 9.71 *μ* (1,030 cm^−1^) appeared to show inverse relationships. Because of the small magnitude of these changes at the latter three wavelengths, statistical correlations based on their rankings were used rather than the actual values, and these were compared with the rankings of durabilities. On the basis of values obtained from spectra of 11 asphalts, Spearman’s rank correlation coefficients were calculated for all four correlations of durability with changes in the value of log 1/*τ* (table 3).

These correlation coefficients are significant at the 1 percent level for 9 degrees of freedom. As for the correlation of durability with changes in transmittance at 5.88 *μ*, a linear relation could be assumed to exist between durability and the reciprocal of changes in log 1/*τ*. The product-moment correlation coefficient was calculated to be 0.779, which is significant at the 1 percent level for the number of samples involved.

## 4. Discussion of Results and Conclusions

A simple technique for studying the structure of whole asphalts by infrared spectroscopy has been developed. The use of this method on 28 asphalts, which come from widely different sources and which vary greatly in durability as roof coatings, showed the asphalts to be remarkably similar in absorption characteristics. In the unexposed asphalt the only absorption band that showed some degree of correlation with durability was that produced by hydroxyl absorption. In general, the less durable asphalts exhibited stronger hydroxyl absorption than the more durable asphalts. The intensities of the band at 7.25 *μ* (1,380 cm^−1^) attributed to C–CH_3_ absorption appeared to show some correlation with durability, but because of variations in film thickness, uncertainties existed which tended to obscure the significance of such a correlation.

The exposure of asphalt films to a carbon arc produced oxidative changes, which were largely the formation of carbonyl and hydroxyl groups and also changes in absorption at 9.71 *μ* (1,030 cm^−1^) and 8.66 *μ* (1,155 cm^−1^). The durabilities of the asphalts showed inverse relationships with changes in absorption at each of these wavelengths. By calculating Spearman’s rank correlation coefficients, each of the correlations was shown to be statistically significant. A product-moment correlation coefficient was calculated for the correlation of durabilities with the changes of log 1/*τ* at 5.88 *μ* (1,700 cm^−1^). This correlation coefficient also indicated that the correlation was highly significant.

The absorption band near 9.71 *μ* (1,030 cm^−1^) was observed in fractions of asphalt by Stewart.^2^ Stewart considered C—O, S=O, or SiO as the most likely groups to be responsible for this absorption. He also observed that weakening of this band in exposed asphaltic fractions appeared to be consistent with oxidation of sulfoxides to sulfones. The asphalt films exposed to the carbon arc in this study, however, showed increases in the band near 9.71 *μ.* The correlation of durabilities with changes in carbonyl and hydroxyl absorption and also with changes in values of log 1/*τ* at 8.66 *μ* (1,155 cm^−1^) and 9.71 *μ* (1,030 cm^−1^) suggest that the changes at these wavelengths may have been caused by changes in concentration of C—O linkages.

## Figures and Tables

**Table 1:** Log 1/τ values obtained from the infrared spectra of asphalt films

Frequency cm^−1^					3,430	2,960	1,700	1,600	1,470	1,380	1,155	1,030	961	866	811	750	720
Wavelength *μ*					2.91	3.38	5.88	6.25	6.80	7.25	8.66	9.71	10.41	11.55	12.33	13.33	13.89
Assigned structural groups					–OH	–CH_2_– –C–CH_3_	C=O	Aromatic	–CH_2_– C–CH_3_	C–CH_3_	…………	C—O, S=O or SiO	Naphthenic	Aromatic substitution			–(CH_2_)_4_–

Asphalt	Softening point a	Penetration at 77° F a	Durability b	Thickness of film	Log 1/*τ* values (determined by base-line method)												
					
	^°^ *F*		*Days*	*Mils*													
E	231	18	23	1.8 to 2.0	0.065	(c)	0.127	0.206	0.948	0.518	0.034	0.021	0.020	0.061	0.066	0.110	0.072
C	230	18	45	1.7 to 1.9	.050	(c)	.059	.153	1.040	.542	.034	.024	.016	.039	.060	.114	.079
G	226	13	51	1.7 to 1.9	.041	(c)	.074	.200	1.040	.478	.042	.026	.019	.096	.068	.116	.080
D	216	12	66	1.8 to 2.1	.019	(c)	.305	.240	1.102	.476	.044	.030	.019	.085	.062	.024	.131
A	220	13	73	1.6 to 1.8	.030	(c)	.071	.171	.942	.415	.035	.012	.017	.078	.070	.117	.100
A	220	13	73	1.6 to 1.8	.032	(c)	.071	.166	.977	.409	.028	.018	.013	.072	.060	.101	.089
B	229	14.5	73	1.5 to 1.9	.025	(c)	.074	.200	1.182	.411	.026	.026	.012	.100	.061	.108	.115
F	221	19	75	1.7 to 2.0	.033	(c)	.036	.192	1.068	.458	.038	.020	.020	.083	.068	.114	.108
F	221	19	75	1.7 to 2.0	.031	(c)	.038	.208	1.064	.480	.036	.014	.020	.082	.076	.123	.100

a Data obtained from asphalt suppliers.

b Durability by accelerated weathering. Data obtained by J. Falzone and S. Ishihara, Research Associates for Asphalt Roofing Industry Bureau, NBS.

c Intense absorption.

**Table 2:** Changes produced in log 1/τ values of asphalts by ultraviolet exposure

Frequency cm^−1^					3,430	1,700	1,600	1,380	1,155	1,030	961	866	811	750	720
WavelengthWavelength *μ*					2.91	5.88	6.25	7.25	8.66	9.71	10.41	11.55	12.33	13.33	13.89
Assigned structural groups					–OH	C=O	Aromatic	C–CH_3_	…………	C—O, S=O, or SiO	Naphthenic	Aromatic substitution			–(CH_2_)_4_–

Asphalt	Softening point a	Penetration at 77° F a	Durability b	Thickness of film	Log 1/*τ* values (determined by base-line method)										
					
	° *F*		*Days*	*Mils*											
Q	227	18	35	2.2 to 2.6	0.055	0.262	0.421	…………	0.036	0.028	0.008	0.071	0.084	0.096	0.101
Q^c^	…………	…………	…………	2.2 to 2.6	.130	.565	.558	…………	.144	.150	.019	.066	.076	.105	.125
R	224	21	38	2.2 to 2.5	.019	.135	.284	0.60	.026	.05	.008	.067	.037	.090	.094
R^c^	…………	…………	…………	2.2 to 2.5	.034	.325	.301	.54	.040	.10	.020	.084	.061	.105	.110
S	222	19	50	2.3 to 2.6	.031	.153	.362	.64	.038	.027	.019	.072	.039	.076	.086
S^c^	…………	…………	…………	2.3 to 2.6	.061	.343	.354	.59	.030	.098	.010	.042	.043	.090	.099
T	223	23	72	2.30 to 2.56	.045	.205	.377	.58	.036	.032	.017	.062	.079	.109	.132
T^c^	…………	…………	…………	2.30 to 2.56	.050	.321	.407	.58	.036	.068	.024	.050	.066	.119	.114

a Data obtained from asphalt suppliers

b Durability by accelerated weathering. Data obtained by J. Falzone and G. McDonald, Research Associates for Asphalt Roofing Industry Bureau, N.B.S.

c Exposed 19.5 hr.

**Table 3:** Correlations of durability with changes in log 1/τ at certain wavelengths

Wavelength (wave number)	Spearman’s rank correlation coefficient
5.88 *μ* (1,700 cm^−1^)	0.782
8.66 *μ* (1,155 cm^−1^)	.945
9.71 *μ* (1,030 cm^−1^)	.836
2.91 *μ* (3,430 cm^−1^)	.815

## Footnotes

1 S. H. Greenfeld, ASTM Bull. 193, 50 (1953).

2 J. E. Stewart, J. Research NBS 58, 265 (1957), RP2759.

3 H. E. Schweyer, Anal. Chem., 30, 205 (1958).

4 ASTM Method D36-26.

5 ASTM Method D5-52.

6 S. H. Greenfeld reported the use of a press for making films with a range of 5 to 50 mils thickness, NBS Building Materials and Structures Report 147 (1956).

7 The symbol *τ* has been used for optical transmittance in accordance with the publication ASA Z 10.6 (1948) of the American Society of Mechanical Engineers.

